# The Potential Role of Butyrate in the Pathogenesis and Treatment of Autoimmune Rheumatic Diseases

**DOI:** 10.3390/biomedicines12081760

**Published:** 2024-08-05

**Authors:** Carmela Coccia, Francesco Bonomi, Anna Lo Cricchio, Edda Russo, Silvia Peretti, Giulia Bandini, Gemma Lepri, Francesca Bartoli, Alberto Moggi-Pignone, Serena Guiducci, Francesco Del Galdo, Daniel E. Furst, Marco Matucci Cerinic, Silvia Bellando-Randone

**Affiliations:** 1 Department of Experimental and Clinical Medicine, Division of Rheumatology, Scleroderma Unit, AOU Careggi, University of Florence, 50139 Florence, Italy; carmela.coccia@unifi.it (C.C.); francesco.bonomi@unifi.it (F.B.); silvia.peretti@unifi.it (S.P.); lepri.gemma@gmail.com (G.L.); francesca.bartoli19@gmail.com (F.B.); serena.guiducci@unifi.it (S.G.); 2 Department of Experimental and Clinical Medicine, Division of Internal Medicine AOUC, University of Florence, 50134 Florence, Italy; anna.locricchio@unifi.it (A.L.C.); giulia.bandini@unifi.it (G.B.); alberto.moggipignone@unifi.it (A.M.-P.); 3 Department of Experimental and Clinical Medicine, University of Florence, 50139 Florence, Italy; edda.russo@unifi.it; 4 Raynaud’s and Scleroderma Programme, NIHR Biomedical Research Centre, Leeds Institute of Rheumatic and Musculoskeletal Medicine, University of Leeds, Leeds LS9 7JT, UK; f.delgaldo@leeds.ac.uk; 5 Department of Rheumatology, University of California Los Angeles, Los Angeles, CA 90095, USA; dan@furst.us.com; 6 Unit of Immunology, Rheumatology, Allergy and Rare Diseases, IRCCS San Raffaele Hospital, 20132 Milan, Italy; matuccicerinic.marco@hsr.it

**Keywords:** microbiota, butyrate, systemic autoimmune diseases, systemic sclerosis, rheumatoid arthritis, short-chain fatty acids, Sjogren’s syndrome, Behçet’s disease, systemic lupus erythematosus

## Abstract

The gut microbiota is a complex ecosystem of microorganisms residing in the human gastrointestinal tract, playing a crucial role in various biological processes and overall health maintenance. Dysbiosis, an imbalance in the composition and function of the gut microbiota, is linked to systemic autoimmune diseases (SAD). Short-chain fatty acids (SCFAs), especially butyrate, produced by the gut microbiota through the fermentation of dietary fibers, play a significant role in immunomodulation and maintaining intestinal homeostasis. Butyrate is essential for colonocyte energy, anti-inflammatory responses, and maintaining intestinal barrier integrity. Studies show reduced butyrate-producing bacteria in SAD patients, suggesting that increasing butyrate levels could have therapeutic benefits. Butyrate’s anti-inflammatory effects and its potential therapeutic role have been studied in rheumatoid arthritis, Sjogren’s syndrome, systemic lupus erythematosus, systemic sclerosis, and Behçet’s disease. Despite promising in vitro and animal model results, human studies are limited, and the optimal strategies for modulating dysbiosis in SADs remain elusive. This review explores the current evidence on the immunoregulatory role of butyrate and its potential therapeutic effects in SAD.

## 1. Introduction

The gut microbiome represents a complex ecosystem of microorganisms that live in the human gastrointestinal (GI) tract, playing a fundamental role in human biological processes and in maintaining individual health. Particularly, the gut microbiota facilitates digestion and synthesizes vitamins; parallelly, another important function is the regulation of the immune system. Indeed, changes in the composition and functions of the gut microbiota induce an imbalanced condition known as “dysbiosis”. This alteration of the bacterial flora impacts pro- and anti-inflammatory immune responses, contributing to the development of various systemic autoimmune diseases (SAD), such as rheumatoid arthritis (RA), vasculitis, systemic lupus erythematosus (SLE), systemic sclerosis (SSc), and Sjogren’s syndrome (SS) [1].

Furthermore, the gut microbiota produces a wide range of metabolites, including short-chain fatty acids (SCFAs). In detail, SCFAs, primarily acetate, propionate, and butyrate, are a subset of fatty acids produced in the intestinal lumen during the bacterial fermentation of non- and partially digestible polysaccharides, specifically resistant starch and dietary fiber and, to a lesser extent, dietary and endogenous proteins. SCFAs play a significant role in immunomodulation and in maintaining intestinal homeostasis by inhibiting the production of pro-inflammatory cytokines, as well as promoting the differentiation of regulatory T (Treg) cells, which are essential for maintaining immune tolerance and preventing inappropriate autoimmune responses [2,3,4,5]. Among SCFAs, butyrate, a four-carbon SCFA, has received particular attention for its beneficial effects on both cellular energy metabolism and intestinal homeostasis [6]. Even though it is not the major SCFAs produced (~60% acetate, 25% propionate, and only 15% butyrate in humans) [7], butyrate is the major energy source for colonocytes [8], yielding around 70% of the cells’ energy. Studies using germ-free animals suggest that butyrate is essentially absent in their GI tracts [9]. This, coupled with the negligible levels of butyrate in our diet, suggests that humans are dependent on the action of the gut microbiota to produce this key metabolite. Butyrate promotes human health in a variety of ways. When activated in colonocyte and immune cells, butyrate causes changes in cytokine levels and various signaling pathways that promote an anti-inflammatory response. In particular, it influences monocytes by increasing IL-10 (an anti-inflammatory cytokine) production and decreasing IL-12 (a pro-inflammatory cytokine) release, further reducing the production of pro-inflammatory molecules such as TNFα, nitric oxide, and IL-1β, as well as suppressing the activity of the transcription factor NF-κB [1,10]. Additionally, butyrate regulates macrophage function [2] and promotes gut mucosa integrity, strengthening the barrier and reducing intestinal permeability, thereby preventing the translocation of pathogenic microorganisms and their products [11,12]. It also regulates histone acetylation by activating, at low concentrations, histone acetyltransferases (HATs) or inhibiting, at high concentrations, histone deacetylases (HDACs) classes I and II; this leads to promotion of Treg differentiation with the respective inhibition of Th17 [13]. Butyrate can also directly influence gene expression by interacting with promoters [14,15]. Moreover, several genes with butyrate response elements have been identified. Different studies have suggested that SCFAs not only have anti-inflammatory actions but could also have beneficial effects on bone formation by modulating the activity and differentiation of osteoclasts, reducing the risk of developing rheumatic diseases, or improving their prognosis [16]. Moreover, *Faecalibacterium*, a butyrate-producer genus, is notably reduced in patients with rheumatic diseases, suggesting that modulation of the gut microbiota through probiotics, or other therapies based on gut microbiota regulation, could be useful in preventing or treating these inflammatory conditions [1]. Investigations into the gut microbiota in patients with SAD have shown a reduction in pro-tolerogenic bacterial species [17,18,19,20]. Pro-tolerogenic bacteria are those that promote immune tolerance, contributing to maintaining balance in the immune system and preventing excessive inflammatory responses. Interestingly, microbiome and metabolome analyses in patients with different types of SADs have revealed common characteristics that may indicate shared etiological factors and pathophysiological mechanisms [21]. Furthermore, a reduction in butyrate-producing bacteria, such as *Lachnoclostridium* and *Lachnospira*, was found in SAD patients compared to healthy controls, suggesting a regulatory and tolerogenic role of SCFAs in immune functions [18,19,22,23]. In addition, metabolomic analysis has shown that SADs have unique characteristics compared to healthy controls, although it has not been possible to identify a unifying discriminatory signature. However, some correlations between intestinal bacterial genera and clusters of metabolites have been identified [24]. For example, a cluster enriched with acylcarnitine metabolites was correlated with bacteria of the genus *Prevotella*, associated with the production of trimethylamine N-oxide from dietary carnitine, which upregulates inflammatory pathways by influencing macrophage activity [25,26]. Conversely, there was an inverse correlation between acylcarnitine and butyrate-producing bacteria, suggesting an association between red-meat-derived carnitine intake and the presence of certain intestinal bacteria [27,28,29,30]. These data indirectly support the idea that modulation of the gut microbiota, whether through diet or other means, could represent a potential strategy to address the systemic consequences of dysbiosis, including inflammation and autoimmunity activation. Currently, the optimal strategy for modulating dysbiosis in SADs remains elusive; however, metabolome analysis appears to be a valid option for monitoring the effect of such intervention [31,32,33,34]. In addition, clinical efforts to increase butyrate levels in humans and reverse possible negative outcomes have generated mixed results. 

On these premises, in this narrative review, we summarize the current evidence on the immunoregulatory action of microbiota-derived butyrate and explore its potential therapeutic effect on rheumatic autoimmune diseases. Specifically, we discuss the role of butyrate in RA, SLE, SSc, SS, and Behçet’s disease (BD).

## 2. Gut Dysbiosis and Systemic Autoimmune Disease (SAD) Development

Analysis of the gut microbiota composition and function in patients with SAD has revealed significant differences compared to healthy controls. Some studies indicate that patients with RA, SS, and SLE exhibit a reduction in the level of *Faecalibacterium*, a butyrate-producing bacterium with anti-inflammatory properties [35], as well as an increase in *Streptococci*, known for their pro-inflammatory effects [36]. So, these alterations suggest a link between gut dysbiosis and systemic inflammation caused by changes in systemic immune responses, a loss of tolerance, and the development of autoimmunity [1,37,38,39]. Moreover, gut dysbiosis can influence the pathogenesis of autoimmune diseases through various biological mechanisms. In particular, intestinal epithelial cells form a barrier that regulates antigen trafficking through paracellular pathways. Microbial dysbiosis can compromise this barrier by stimulating zonulin (a protein that increases the permeability of tight junctions) production and thus increasing intestinal permeability. These processes allow microbial fragments to translocate into the lamina propria [40,41,42,43]. Here, microbial fragments can bind to specific receptors and activate pro-inflammatory T cells (T helper 1 and 17), which stimulate B cells to produce autoantibodies. These activated immune cells can then migrate to other organs and tissues, exacerbating systemic inflammation and thus contributing to the development of rheumatic diseases [4,44,45,46,47,48,49,50].

## 3. Diseases Listed Alphabetically (List of Studies Analyzed and the Main Findings Are Shown in Table 1, Different Mechanisms of Action Are Summarized in Figure 1)

### 3.1. Behçet’s Disease (BD)

BD is a recurring multisystemic inflammatory disease typically characterized by oral and genital aphthous ulcers, as well as skin and ocular lesions. The disease can also present with vascular, articular, neurological, and GI symptoms [51]. A systematic review of the microbiome profiles in BD underlines the presence of gut dysbiosis in patients [52], characterized by a reduction in butyrate-producing bacteria. In a study conducted in vitro, Yun et al. [53] focused on butyrate’s action, demonstrating that this metabolite suppresses inflammatory cytokine production (IL-6, TNF-α, and IL-1β) in peripheral blood mononuclear cells (PBMCs) isolated from patients with mucocutaneous involvement of BD and healthy controls. The anti-inflammatory effect of butyrate was greater in the BD patients, so the authors analyzed the butyrate receptor expression in both groups and found that the mRNA levels of the lipopolysaccharide-induced free fatty acid receptor 2 in the PBMCs were higher of the BD patients than those of the healthy controls. In addition, in a clinical study involving BD patients, butyrate-rich diets were administered for 3 months (a habitual diet supplemented with oral butyrate (2.4 g/day) versus a lacto-ovo-vegetarian diet containing inulin and resistant starch-rich foods, whose fermentation increases butyrate production). After 3 months, in both groups, the authors observed a reduction in leukocyte oxygen-containing reactive species (ROS) production and plasma lipid peroxidation, an increase in plasma total antioxidant capacity, and a significant improvement in fibrin susceptibility to plasmin-induced lysis. These findings suggest the benefits of a butyrate-rich diet for cardiovascular disease prevention in BD. Simultaneously, BD disease activity decreased in both groups, with a consequential reduction in corticosteroid use. However, the authors did not observe a significant improvement in their blood inflammatory parameters or changes in their gut microbiota or SCFAs, suggesting that longer dietary interventions are needed to affect microbial resilience [54]. This research has highlighted the potential use of butyrate for inflammation control in BD patients, but further studies in vivo are required.

**Figure 1:**
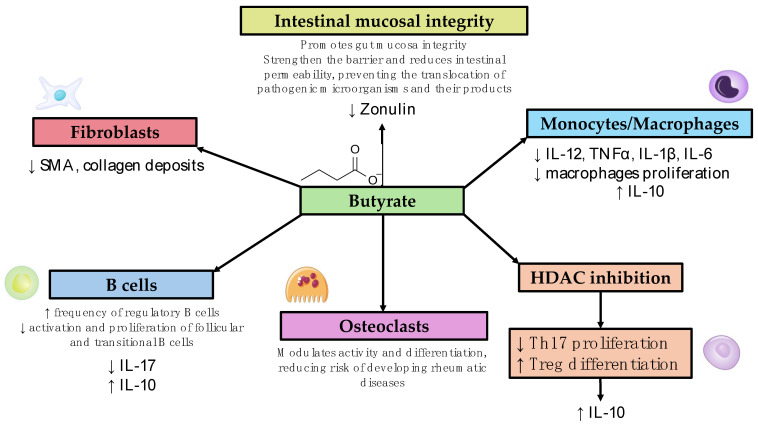
Mechanism of action of butyrate and its role in modulating the immune system. HDAC: histone deacetylase, SMA: smooth muscle actin.

### 3.2. Rheumatoid Arthritis (RA)

RA is a chronic autoimmune disorder characterized by inflammation in multiple organs but with prominent joint involvement, leading to joint pain, swelling, and potential joint destruction. Studies indicate that gut microbiota dysbiosis may contribute to RA pathogenesis by altering immune responses and promoting systemic inflammation. In the context of RA, butyrate’s impact has been particularly noteworthy. Indeed, recent studies have highlighted the significance of the gut microbiota and its metabolites, especially butyrate, in modulating immune responses and inflammation associated with RA [55]. Dysbiosis has been observed in RA patients, highlighting a reduction in butyrate-producing bacteria such as those belonging to the *Clostridium* cluster XIVa [56]. This type of dysbiosis may contribute to the pathophysiology of RA by enhancing systemic inflammation. Additionally, studies have shown that new-onset untreated RA is associated with an underrepresentation of butyrate-producing species and an overwhelming presence of butyrate consumers. Therefore, this condition suggests the possibility of an important role for butyrate in maintaining immune homeostasis and its potential therapeutic application in RA. Moreover, this abovementioned gut microbiota imbalance is more pronounced in patients with positive anti-citrullinated protein antibodies [57]. As previously noted, butyrate has notable immunomodulatory effects, acting on various immune cells, including Tregs, macrophages, and dendritic cells [58]. In RA, butyrate promotes the differentiation and function of Tregs, which are essential for maintaining immune tolerance. The net butyrate yield, defined as the ratio between producers and consumers, showed a negative correlation with disease activity, antibody production, and joint deformity, highlighting the beneficial effect of butyrate in RA. Interestingly, fecal levels of butyrate were shown to correlate with its serum levels, negatively correlating with the number of deformed joints in RA patients. It enhances the production of IL-10, an anti-inflammatory cytokine, within Tregs, thereby inhibiting the pro-inflammatory cytokines secreted by T helper 17 (Th17) cells [57]. Additionally, butyrate increases the frequency of regulatory B cells (Bregs) and reduces the activation and proliferation of follicular and transitional B cells [59]. Several experimental studies utilizing mouse models of RA have provided compelling evidence of butyrate’s benefits. In these animal models, dietary butyrate supplementation significantly mitigated the onset and severity of arthritis symptoms [57]. For example, butyrate reduced the serum levels of zonulin and alleviated inflammation-driven shortening of the small intestine; it is known, in fact, that permeation of the inflammatory cells and edema of the intestinal wall may lead to the contraction of the small and large intestines’ length [44]. Moreover, in collagen-induced arthritis models, butyrate suppressed autoimmune arthritis development by promoting follicular regulatory T (TFR)-cell differentiation [56]. It achieves this by enhancing histone acetylation through histone deacetylase inhibition [58], which increases the expression of TFR-cell marker genes. The adoptive transfer of butyrate-treated TFR cells reduced type-2-collagen-specific autoantibody production and ameliorated arthritis symptoms, indicating butyrate’s role in enhancing the TFR cells to suppress autoantibody production [58]. Gut microbiota dysbiosis in RA patients is linked to increased intestinal permeability, which facilitates the transition from asymptomatic autoimmunity to inflammatory disease. Zonulin is highly expressed in autoimmune conditions, and its levels can predict the onset of inflammatory arthritis [44]. In fact, increased serum zonulin levels lead to a leaky intestinal barrier, gut dysbiosis, and inflammation. In mouse models of pre-phase arthritis, restoring the intestinal barrier using butyrate or a cannabinoid type 1 receptor agonist was shown to inhibit arthritis development [44]. Treatment with larazotide acetate, a zonulin antagonist that enhances intestinal barrier integrity, was shown to effectively reduce arthritis onset, highlighting a preventive approach to autoimmune diseases by targeting intestinal barrier function [44]. Studies have shown that butyrate supplementation can enrich beneficial bacterial populations in the gut, increasing the abundance of Tregs in the systemic circulation [58]. This modulatory activity of butyrate helps control local gut inflammation and induces systemic effects that are able to reduce the overall severity and progression of RA. So, modifying the gut microbiota to increase the prevalence of butyrate-producer species and simultaneously decrease butyrate consumption could potentially lead to a higher level of butyrate production, ultimately improving inflammatory conditions and promoting a more positive outlook.

Recent research has also focused on improving the bioavailability of butyrate. The development of butyrate as a drug is particularly difficult due to its poor oral bioavailability, as it is rapidly metabolized in the gut and has low potency (hence, necessitating high dosing). A recent study analyzed a new form of butyrate, esterified into serine (creating SerBut), showing how this enhanced its systemic uptake by aiding its escape from the gut [60]. In mouse models of collagen-induced arthritis, SerBut substantially ameliorated disease severity, modulated key immune cell populations, and reduced inflammatory responses [61].

### 3.3. Sjogren’s Syndrome (SS)

SS is an autoimmune disease typically characterized by chronic inflammation of exocrine glands, with possible involvement of internal organs. The disease is derived from the T-cell-mediated hyperactivation of B cells with inflammatory cytokines and autoantibody production. Recent studies have extensively documented the changes in microbial communities in the intestinal, oral, and ocular regions due to SS, though some of these findings have been inconsistent. Additionally, dysbiosis is linked to the worsening of symptoms and increased disease activity in SS patients. However, the role of the microbiome in the pathogenesis of SS, whether as a cause or effect, remains unclear. As recently described by Wang et al., the composition and function of the gut microbiota were significantly different in SS patients compared with healthy controls [62]. Interestingly, recent studies have shown that a lower *Firmicutes*-to-*Bacteroidetes* (F/B) ratio is a key characteristic of the gut microbiota in SS patients in comparison to healthy controls [63]. This implies that *Firmicutes* are suppressed, while *Bacteroidetes* become conditional pathogens, indicating the presence of gut microbial dysbiosis in SS. A study published by Kim et al. [64] emphasizes the effect of butyrate in a murine model of SS. After the intraperitoneal administration of butyrate (which makes the application of these data to humans highly problematic), there was an increase in the salivary flow rate and histologically a reduction in inflammation of the salivary glands. In an in vitro model using human submandibular gland cells, butyrate induced IL-10-producing B cells and decreased IL-17-producing B cells [64]. In SS, butyrate is the most frequently cited bacterial SCFA. Numerous studies have reported that butyrate-producing bacteria, such as Faecalibacterium prausnitzii, Bacteroides fragilis, Lachnoclostridium, Roseburia, Lachnospira, and Ruminococcus, are significantly reduced in SS patients [63,64]. Furthermore, recent research has highlighted the role of butyrate-producing bacteria, Bacteroides spp., and Clostridia clusters XIVa and IV in maintaining Treg/Th17 balance [65]. It is important to note that the differentiation of Treg cells is promoted not only by butyrate but also by the immunomodulatory molecule polysaccharide A from Bacteroides fragilis. Disrupting the Treg/Th17 balance can compromise the mucosal barrier’s protection against pathogenic microorganisms [65]. Butyrate can influence T-cell balance and regulate the frequency of IL-10- and/or IL-17-producing B cells by modulating circadian-clock-related genes to perform anti-inflammatory functions. Additionally, butyrate can increase the flow rate of saliva and alleviate salivary gland inflammation. These findings suggest that reduced SCFAs or butyrate-producing bacteria might impact the permeability of the mucosal barrier, the frequency or function of immune cells, and even salivary gland secretion.

### 3.4. Systemic Lupus Erythematosus (SLE)

SLE is a systemic autoimmune disease associated with the activation of both innate and adaptive immunity. This leads to the production of autoantibodies and immune complexes, leading to inflammation and tissue damage [66]. Recent studies have focused their attention on the pathogenetic role of the intestinal microbiota in autoimmune diseases, including SLE [67,68]. A cross-sectional study demonstrated a significant reduction in butyrate-producing bacteria, with a decrease in the *Firmicutes/Bacteroidetes* ratio in SLE patients compared to healthy subjects [69]. A microbiome analysis in SLE patients revealed a decrease in intestinal microbial diversity and altered SCFA production [18]. Only a few studies have focused on the immunoregulatory function of SCFAs and butyrate in SLE patients. He et al. evaluated the effect of butyrate on the intestinal microbiota and renal involvement in a lupus-like mouse model. Their results showed an improvement in renal damage, with a lower degree of mesangial cell proliferation, less mesangial matrix widening, and reduced inflammatory cell infiltration and interstitial fibrosis in lupus-like mice after treatment with butyrate. Further, after 8 weeks of butyrate supplementation, there was an improvement in intestinal dysbiosis, with an increase in *Firmicutes* and *Clostridia* and a reduction in *Bacteroidetes* [70]. In another study, Sanchez et al. demonstrated the interesting ability of butyrate to reduce the levels of specific SLE autoantibodies (anti-dsDNA, anti-RNP/Sm, anti-RNA, and anti-histone) and plasma cell differentiation in lupus-like mice [71]. Some studies have focused on the role of the gut microbiota, in particular butyrate, as potentially having a protective effect on cardiovascular manifestations in SLE patients [72,73]. Two different mouse studies on SLE showed a beneficial effect of butyrate on blood pressure, left ventricular hypertrophy, vascular oxidative stress, Th17-cell infiltration in the aorta, and endothelial dysfunction [74,75]. This was demonstrated in one study [74] by directly supplementing sodium butyrate (40 mM in drinking water for 8 weeks) and in the other [75] by supplementing fiber able to increase butyrate-producing bacteria (250 mg of soluble fiber/mouse/day in drinking water). In a randomized, double-blind, placebo-controlled trial, the authors investigated whether synbiotic supplementation (capsules containing 3 × 10^9^ colony forming units (CFU) of *Lactobacillus helveticus* R0052 60%, *Bifidobacterium infantis* R0033 20%, and *Bifidobacterium bifidum* R0071 20%) for 60 days could restore the balance of the gut microbiota and reduce systemic inflammation in SLE patients. This study involved 46 patients with SLE and GI symptoms (23 randomly assigned to the synbiotic group and 23 to the control group). The synbiotic supplementation was able to modify the gut microbiota in the synbiotic group, with an increase in butyrate-producing bacteria and butyrate metabolism. These patients demonstrated a significant reduction in serum IL-6 levels, suppression of an increase in high-sensitivity C-reactive protein (hs-CRP) levels, and an improvement in their SLE disease activity scores as measured by SLEDAI-2K [76]. Larger trials with longer synbiotic treatments are needed to confirm these results, and further studies are imperative to better understand the role and potential therapeutic effect of butyrate in SLE patients.

### 3.5. Systemic Sclerosis (SSc)

SSc is a systemic connective disease characterized by chronic inflammation, vasculopathy, and fibrosis involving the skin and internal organs [77]. Gastrointestinal involvement is present in almost all SSc patients [78,79]. Recent studies have focused on the association between dysbiosis and the pathogenesis and progression of SSc, recognizing the gut microbiota as a promising therapeutic target, especially for GI involvement [80,81,82]. Dysbiosis found in SSc patients is characterized by a reduction in protective butyrate-producing bacteria and by an increase in pro-inflammatory noxious genera [20]. An anti-fibrotic effect of butyrate was demonstrated by Kabel et al. in an idiopathic pulmonary fibrosis (IPF) mouse model [83]. In a single study, the administration of sodium butyrate (10 mg orally five times a week over four weeks or 2 mg subcutaneously five times a week for two weeks) in a bleomycin mouse model with dermal and lung fibrosis reduced the expression of alpha-smooth muscle actin (SMA) in the skin (a myofibroblast marker), reduced collagen deposits and skin thickness, suppressed macrophage proliferation, reduced the expression of profibrotic and pro-inflammatory genes in the fibrotic skin, and reduced SMA protein in the fibrotic lung tissue [84]. In a recent study comparing the fecal microbiota and SCFAs between patients with very early SSc (VEDOSS) and those with established SSc, researchers identified gut microbiota dysbiosis in VEDOSS patients for the first time. This dysbiosis was marked by a reduction in the probiotic/protective anti-inflammatory intestinal flora, which mainly comprises butyrate-producing bacteria [85]. Moreover, VEDOSS patients exhibited a significant reduction in fecal butyrate. This early gut microbiota imbalance may promote the growth of pro-inflammatory harmful microbiome members, thereby worsening intestinal dysbiosis and inflammation from the disease’s earliest phases. These findings suggest that further clinical research is needed to explore whether administering butyrate in the very early stages of the disease could serve as a new therapeutic strategy to slow disease progression and alleviate symptoms, ultimately enhancing patient quality of life. However, these are early preliminary results that should be repeated and extended. Unfortunately, there are no studies regarding the potential therapeutic effect of butyrate on GI involvement in SSc.

**Table 1:** List of Studies Analyzed and Main Findings About Role of Butyrate in Autoimmune Rheumatic Diseases.

Authors	Year of Publication	Type of Study	Main Findings
**Behçet’s Disease**			
Yun et al. [53]	2018	In vivo Cross-sectional study on 11 patients with Behçet’s disease and 10 healthy controls	- Butyrate suppresses inflammatory cytokine production (IL-6, TNF-α, and IL-1β) in peripheral blood mononuclear cells (PBMCs) isolated from patients with mucocutaneous involvement of BD and healthy controls - mRNA levels of the lipopolysaccharide-induced free fatty acid receptor 2 in the PBMCs were higher of BD patients than in healthy controls
Emmi et al. [54]	2021	In vivo Proof-of-concept randomized trial study on 17 patients with Behçet’s disease	- A butyrate-rich diet may be beneficial in terms of cardiovascular risk in BD patients - A butyrate-enriched diet led to decreased disease activity
**Rheumatoid Arthritis**			
He J et al. [57]	2022	Mice model	- Underrepresentation of butyrate-producing species was associated with new-onset untreated RA - Butyrate increases the production of IL-10 within Tregs, inhibiting the pro-inflammatory cytokines secreted by Th17 - Butyrate promotes the differentiation and function of Tregs - Dietary butyrate supplementation improved symptom burden and disease severity
Kim DS et al. [58]	2018	Mice model	- Butyrate was displayed to have immunomodulatory effects (acting on Tregs, macrophages, dendritic cells) - Butyrate supplementation can enrich beneficial bacterial populations in the gut, increasing the abundance of Tregs in the systemic circulation
Rosser EC et al. [59]	2020	Mice model/in vitro	- Butyrate increases the frequency of Bregs - Reduces the activation and proliferation of follicular and transitional B cells
Tajik N et al. [44]	2020	Mice model	- Butyrate reduces levels of zonulin, improving gut inflammation - Butyrate supplementation was able to restore the intestinal barrier, inhibiting arthritis development
Takahashi D et al. [56]	2020	Mice model	- Butyrate reduces risk of arthritis development by promoting follicular Treg-cell differentiation - Decreased autoantibody production by enhancing follicular Tregs
Balakrishnan B et al. [61]	2021	Mice model	- Butyrate substantially ameliorated disease severity, modulated key immune cell populations, and reduced inflammatory responses
**Sjogren’s Syndrome**			
Kim DS et al. [64]	2021	Mice mode/in vitro	- Administration of butyrate lead to an increase in the salivary flow rate and histologically a reduction in inflammation of the salivary glands - Butyrate induced IL-10-producing B cells and decreased IL-17-producing B cells
Moon J et al. [86]	2020	Cross-sectional study on 10 pSS patients, 14 subjects with dry eye symptoms, and 12 healthy controls	- Butyrate-producing bacteria, including *Faecalibacterium prausnitzii*, *Bacteroides fragilis*, *Lachnoclostridium, Roseburia, Lachnospira*, and *Ruminococcus*, are significantly decreased in SS patients
Cano-Ortiz et al. [87]	2020	Cross-sectional study on 19 pSS patients compared to 19 healthy controls	- The pSS patients had less beneficial or commensal butyrate-producing bacteria and a higher proportion of opportunistic pathogens with pro-inflammatory activity compared to healthy controls.
**Systemic Lupus Erythematosus**			
He et al. [70]	2020	Mice model	- Significant reduction in butyrate-producing bacteria with a decrease in the *Firmicutes/Bacteroidetes* ratio in SLE patients compared to healthy subjects - Effect of butyrate on intestinal microbiota and renal involvement in a lupus-like mouse model. Their results showed an improvement in renal damage with a lower degree of mesangial cell proliferation, less mesangial matrix widening, and reduced inflammatory cell infiltration and interstitial fibrosis in lupus-like mice after treatment with butyrate. Further, after 8 weeks of butyrate supplementation, there was an improvement in intestinal dysbiosis, with an increase in *Firmicutes* and *Clostridia* and a reduction in *Bacteroidetes.*
Sanchez et al. [71]	2020	Mice model	- Ability of butyrate to reduce the levels of specific SLE autoantibodies (anti-dsDNA, anti-RNP/Sm, anti-RNA, and anti-histone) and plasma cell differentiation in lupus-like mice
Moleòn J et al. [74]; Moleòn et al. [75]	2023; 2023	Mice model	- Beneficial effect of butyrate on blood pressure, left ventricular hypertrophy, vascular oxidative stress, Th17-cell infiltration in the aorta, and endothelial dysfunction
Widhani A et al. [76]	2022	Randomized, double-blind, placebo-controlled trial on 23 patients per group	- The synbiotic supplementation was able to modify the gut microbiota in the synbiotic group with an increase in butyrate-producing bacteria and butyrate metabolism. These patients demonstrated a significant reduction in serum IL-6 levels, suppression of the increase in high-sensitivity C-reactive protein (hs-CRP) levels, and an improvement in SLE disease activity scores as measured by SLEDAI-2K.
**Systemic Sclerosis**			
Park HJ et al. [84].	2021	Mice model	- Administration of butyrate reduced the expression of alpha smooth muscle actin (SMA) in the skin (a myofibroblast marker), reduced collagen deposits and skin thickness, suppressed macrophage proliferation, reduced the expression of profibrotic and pro-inflammatory genes in fibrotic skin, and reduced SMA protein in fibrotic lung tissue
Russo E et al. [85]	2024	Cross-sectional study on 26 patients	- Gut dysbiosis with a reduction in butyrate-producing bacteria and a significant decrease in fecal butyrate in patients with very early SSc (VEDOSS) compared to healthy controls

## 4. Conclusions and Future Perspective

The current research has focused on the capacity of butyrate to enhance immune tolerance and to regulate the gut microbiota’s anti-inflammatory and immunoregulatory properties, thus alleviating rheumatic disease symptoms and reducing systemic inflammation in vitro and in animal models. However, at present, data on humans affected by systemic autoimmune diseases are derived from few patients. Moreover, the bioavailability of butyrate and dose issues in humans still remain to be addressed. Therefore, more work needs to be undertaken to elucidate the role that the gut microbiota and butyrate may have in modulating the immune system and restoring gut homeostasis. In the future, new data might open up new avenues for treatment strategies based on the modulation of the gut microbiota, potentially with the help of butyrate.
